# Sustainability in Radiology: Position Paper and Call to Action From ACR, AOSR, ASR, CAR, CIR, ESR, ESRNM, ISR, IS3R, RANZCR, and RSNA

**DOI:** 10.1111/1754-9485.13842

**Published:** 2025-02-22

**Authors:** Andrea G. Rockall, Bibb Allen, Maura J. Brown, Tarek El‐Diasty, Jan Fletcher, Rachel F. Gerson, Stacy Goergen, Amanda P. Marrero González, Thomas M. Grist, Kate Hanneman, Christopher P. Hess, Evelyn Lai Ming Ho, Dina H. Salama, Julia Schoen, Sarah Sheard

**Affiliations:** ^1^ Department of Surgery and Cancer, Faculty of Medicine Imperial College London London UK; ^2^ Department of Radiology Imperial College Healthcare NHS Trust London UK; ^3^ President International Society of Radiology Reston Virginia USA; ^4^ Department of Radiology Grandview Medical Center Birmingham Alabama USA; ^5^ Department of Radiology, Faculty of Medicine University of British Columbia Vancouver British Colombia Canada; ^6^ Diagnostic Imaging BC Cancer Vancouver British Columbia Canada; ^7^ Radiology department, Urology and Nephrology Center University of Mansoura Mansoura Egypt; ^8^ Egyptian Society of Radiology and Nuclear Medicine (ESRNM) Cairo Egypt; ^9^ Monash Health Clayton Victoria Australia; ^10^ Northwest Radiologists Bellingham Washington USA; ^11^ Monash University Clayton Victoria Australia; ^12^ Department of Diagnostic Radiology University of Puerto Rico School of Medicine San Juan Puerto Rico; ^13^ University of Wisconsin Madison Wisconsin USA; ^14^ Department of Medical Imaging University of Toronto Toronto Ontario Canada; ^15^ Joint Department of Medical Imaging, University Health Network (UHN) and Sinai Health System (SHS) University Medical Imaging Toronto Toronto Ontario Canada; ^16^ Department of Radiology and Biomedical Imaging University of California San Francisco California USA; ^17^ ParkCity Medical Centre Kuala Lumpur Malaysia; ^18^ Radiology and Medical Imaging Technology Department Misr University for Science and Technology October City Egypt; ^19^ University of Michigan Ann Arbor Michigan USA

**Keywords:** climate change, health services accessibility, radiology, resource allocation, sustainability

## Abstract

The urgency for climate action is recognised by international government and healthcare organisations, including the United Nations (UN) and World Health Organisation (WHO). Climate change, biodiversity loss, and pollution negatively impact all life on earth. All populations are impacted but not equally; the most vulnerable are at highest risk, an inequity further exacerbated by differences in access to healthcare globally. The delivery of healthcare exacerbates the planetary health crisis through greenhouse gas emissions, largely due to combustion of fossil fuels for medical equipment production and operation, creation of medical and non‐medical waste, and contamination of water supplies. As representatives of radiology societies from across the globe who work closely with industry, and both governmental and non‐governmental leaders in multiple capacities, we advocate together for urgent, impactful, and measurable changes to the way we deliver care by further engaging our members, policymakers, industry partners, and our patients. Simultaneous challenges including global health disparities, resource allocation, and access to care must inform these efforts. Climate literacy should be increasingly added to radiology training programmes. More research is required to understand and measure the environmental impact of radiological services and inform mitigation, adaptation and monitoring efforts. Deeper collaboration with industry partners is necessary to support innovations in the supply chain, energy utilisation, and circular economy. Many solutions have been proposed and are already available, but we must understand and address barriers to implementation of current and future sustainable innovations. Finally, there is a compelling need to partner with patients, to ensure that trust in the excellence of clinical care is maintained during the transition to sustainable radiology. By fostering a culture of global cooperation and rapid sharing of solutions among the broader imaging community, we can transform radiological practice to mitigate its environmental impact, adapt and develop resilience to current and future climate and environmental threats, and simultaneously improve access to care.

**Abbreviations**
AdvaMed: Advanced Medical Technology Association
COCIR: European Coordination Committee of the Radiological, Electromedical and Healthcare IT Industry
EPA: Environmental Protection Agency
HVAC: heating, ventilation and air‐conditioning
IAEA: International Atomic Energy Agency
LMICs: low‐ and middle‐income countries
MEPA: Medical Equipment Proactive Alliance for Sustainable Healthcare
UN: United Nations
WHO: World Health Organisation

## 1 Recognising the Environmental Impact of Radiology and Need for Advocacy

Sustaining a healthy planet is essential to human health, including individual, community and global health [1]. The planetary health framework recognises that the health of all living species, including humans, is interconnected with non‐living components in the natural world [2]. The environment in which we live directly impacts human health, including climate and weather patterns. Human activities, predominantly the burning of fossil fuels, increase greenhouse gases, driving anthropogenic climate change. As healthcare professionals, we can protect our patients by responding to the health effects of climate change and other environmental exposures and signal the importance of an urgent transition to environmentally sustainable imaging practice [3]. The delivery of healthcare generates substantial greenhouse gas emissions, with diagnostic services accounting for approximately 9% of healthcare emissions in one region in Australia [4]. Globally, CT and MR imaging were estimated to contribute up to 0.77% of total carbon dioxide emissions for 2016 with an expected 30% growth between 2016 and 2030 [5, 6]. For comparison, aviation contributed an estimated 2.5% worldwide in 2023 [7].

Transformation of medical imaging to low carbon and climate resilient systems will require engagement at all organisational levels locally and globally, and at a societal level. Individual radiologists and radiology practices can act as agents for change in their daily practice and within their healthcare organisations. Radiological societies may contribute by influencing not only our community but government policy. We must consider the environmental impact of our daily work, balancing responsible imaging usage against the immediate needs of patients. Despite the challenges, sustainable value‐based imaging is possible, with many innovative technologies at our fingertips.

**FIGURE 1:**
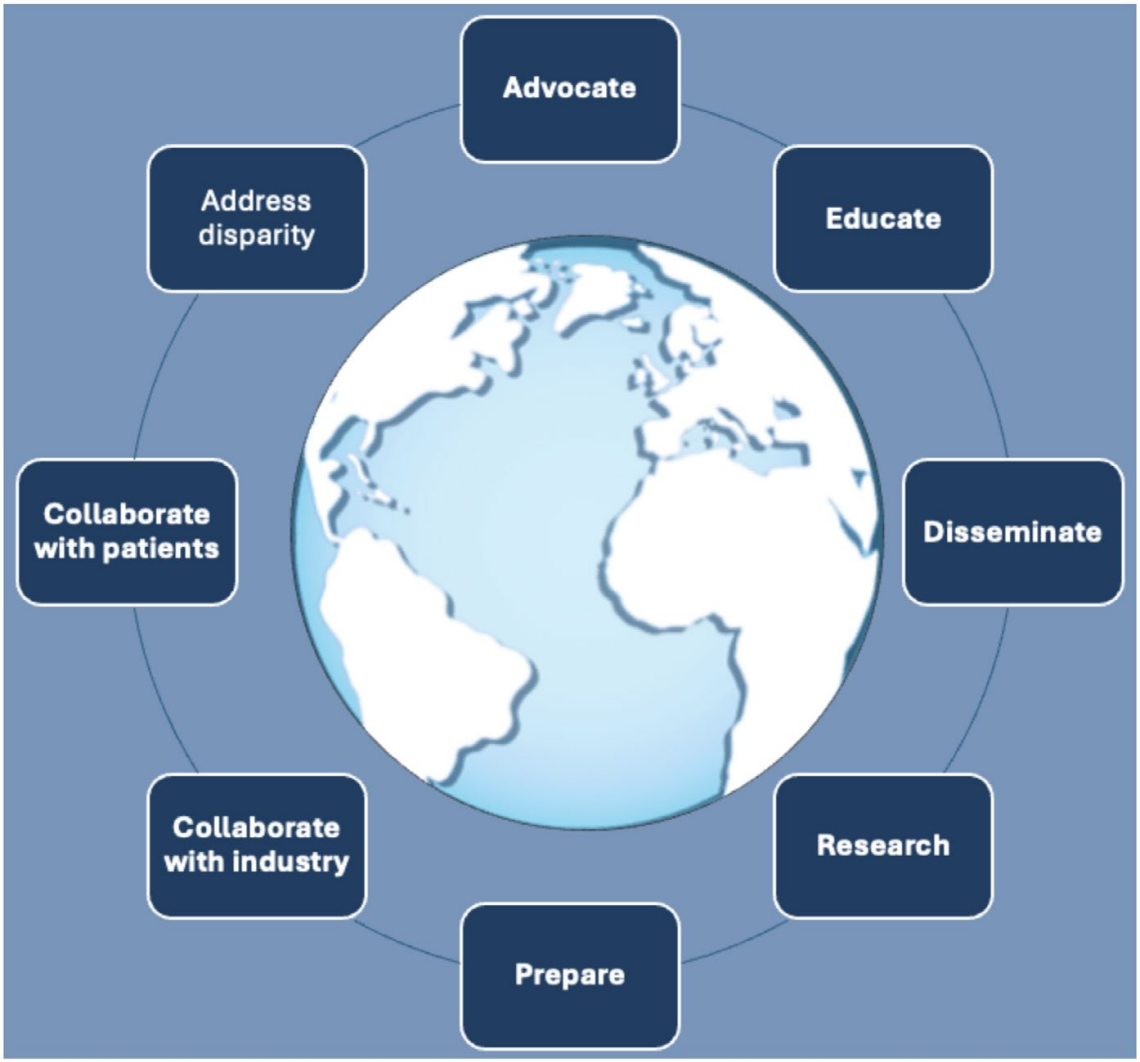
Key actions for radiological societies.

What are the priority actions that professional societies can take? What actions are likely to have the largest impact, require the lowest effort and encounter the fewest barriers to implementation? Eight priority actions are enumerated in this paper, and an action framework has been developed, identifying the responsibilities, impacts and accountability (Figure 1 and Table 1).

**TABLE 1:** Key actions for radiology leadership and societies.

Action	Responsibilities	Expected impact	Responsible parties
Advocacy	Engage in policy discussions and represent radiology interests	Increased influence on healthcare strategies and improved policy outcomes for sustainability	Radiological societies Advocacy groups
Address global disparities	Identify and support initiatives that target disparities	Improved access to sustainable radiological care for underserved populations	Radiological societies Non‐governmental organisations Governmental bodies
Educating for climate literacy	Develop educational materials and programs and make widely available	Enhanced understanding of sustainability among radiology professionals and students	Radiological societies Educational institutions Professional societies
Toolkit	Create and disseminate resources and training modules to support departmental change	Empowerment of radiology departments to adopt sustainable practices effectively	Radiological societies Training organisations
Research	Fund and conduct studies on energy and waste metrics Prioritise research into innovative solutions to the environmental impacts of medical imaging	Evidence‐based insights leading to effective sustainability measures in radiology Empowerment of researchers in academia and industry to pursue innovative research into environmental mitigations	Radiological societies Society publications Research institutions Funding agencies
Preparedness	Develop guidelines for climate emergency responses	Enhanced resilience of radiology departments to climate‐related disruptions and health impacts	Radiological societies Disaster response agencies
Industry collaboration	Partner with manufacturers to create common metrics for emissions and waste Create an examination/procedure ‘energy report’ analogous to radiation ‘dose report’	Adoption of circular economy principles, reducing emissions, waste, and improving supply chain sustainability Energy reporting could contribute to a further visualisation of the problem	Radiological societies Industry partners
Patient collaboration	Engage with patient advocacy groups for feedback	Sustainable patient pathways that maintain high‐quality care and satisfaction	Radiological societies Patient advocacy groups

## 2 Work to address global disparities in impact of climate change

Social, economic and geographical determinants of health, health access, and equity are inextricably linked to environmental sustainability. While no one is immune to the effects of climate change, the climate crisis disproportionately affects low‐resource settings and vulnerable individuals and groups, potentially widening existing health disparities. Extreme weather, heat stress, exposure to wildfire smoke and poor air quality increase the risk of adverse health outcomes including myocardial infarction, asthma exacerbations, injury, zoonotic disease and stroke [8]. People at the extremes of age, individuals with pre‐existing chronic disease, those living in poverty, with food and housing insecurity, or with limited healthcare access are at higher risk of adverse outcomes related to climate disruptions [9]. Strategic action is needed to ensure equitable access to radiology globally, in low‐income settings but also in high income countries with internal disparities in access to care. This will require building capacity while minimising the detrimental environmental effects related to the delivery of radiology services.

In low‐ and middle‐income countries (LMICs), the principal challenges are infrastructure, logistics and human resources, which are made more challenging in sparsely populated regions [7]. Due to limited access to care, health services generate a much lower environmental impact in these countries compared with their middle‐ and higher‐income counterparts. The desire to maintain a low carbon footprint should not impede advances in healthcare that improve overall health in these regions. The current relative lack of medical imaging infrastructure in LMICs presents an opportunity to implement coordinated, sustainable systems [8], such as building reliable renewable energy sources and using mobile equipment [10]. Particular consideration needs to be given to the lifespan and maintenance of equipment [11]. The use of digital tools and remote access options, such as teleradiology and on‐line consultations, mobile units and community outreach can increase accessibility while reducing emissions from patient and staff travel and transportation [6, 12].

LMIC‐led research is essential to provide constructive and locally applicable recommendations. Population and community‐based monitoring of disease and climate trends as well as co‐created educational and screening initiatives will be necessary to simultaneously address climate impacts and ensure access to care.

As access to radiology services in underserved regions across the globe is improved, environmentally sustainable infrastructure should be a cornerstone of government and department policy [13, 14, 15, 16, 17, 18, 19]. Collaborations between international agencies, including the World Health Organisation (WHO) and International Atomic Energy Agency (IAEA), professional organisations, industry and local leadership are critical to increase awareness and influence policies [20].

## 3 Fostering Research

Recent decades have seen the development of a robust body of science about climate change and its impacts on health and society. However, research that specifically elaborates the relationship between medical imaging and its impact on environmental sustainability is only nascent. These associations must be defined across the imaging value chain to more effectively steward energy utilisation and to foster the incorporation of more renewable energy sources in the delivery of imaging‐based healthcare.

Among the more pressing research needs are measurements and benchmarks that foster environmental impact reduction. Efforts to date have focused on life cycle assessment [21] or on energy consumption of specific imaging devices in the operational phase [22, 23] to guide the development of more efficient per‐instrument power management strategies. However, work is required to design data‐driven models of energy consumption, not only for individual imaging devices or examinations but also at a larger scale, for example, within an imaging facility, across a fleet of imaging devices and across a patient's care journey from initial diagnosis through later treatment and disease monitoring [24]. Research into the environmental impact of development and utilisation of AI in radiology, with its enormous usage of energy for model training and foundation models, is essential [25].

Furthermore, there is a compelling need to deliver imaging services more efficiently within large, complex healthcare systems. Decreasing the energy consumption of imaging requires reducing the need for patient and employee travel to monolithic healthcare facilities. Research to develop technology that allows remote operation of imaging devices, deployment of imaging devices more broadly across communities and more appropriate use of imaging resources [26] will synergistically enhance energy mitigation strategies.

Transformation to sustainable practice is, in essence, a study in change management. There is a need for research that compares various approaches to communicating sustainability and their impact on changing behaviours. This is perhaps not a traditional topic of research for radiologists, and we will need new collaborations to bring strength to this area of research.

A major obstacle to reducing imaging's environmental footprint is the lack of public funding mechanisms to support research on the topic. Nearly all research on the environmental sustainability of imaging to date has been unsupported or supported at a small scale by academic departments and by industry.

## 4 Industry Collaboration

Radiology has a long and productive history of innovation through academic‐industry partnerships. The advanced imaging technique resulting from these collaborations have transformed medical practice. Together, we stand uniquely positioned to lead the effort to reduce the environmental impact of medical imaging and improve human health. Radiology and industry leaders need to work with industry groups such as the European Coordination Committee of the Radiological, Electromedical and Healthcare IT Industry (COCIR), the Advanced Medical Technology Association (AdvaMed), the Medical Equipment Proactive Alliance for Sustainable Healthcare (MEPA) and governmental regulatory agencies like the US Environmental Protection Agency (EPA) to establish common metrics and recognition of compliance with sustainability goals [27, 28]. For example, COCIR efforts have helped to establish commonly accepted measurements of energy consumption for medical imaging devices, and the EPA is in the process of developing an Energy Star program to recognise and encourage efforts to reduce energy consumption, likely beginning with MRI [27, 29]. Finally, industry and the radiology community can partner to develop new methods to reduce energy consumption and resource use in medical imaging through collaborative research supported by governmental agencies [30]. For example, equipment manufacturers, contrast media suppliers and radiology researchers may work together to develop AI‐based MRI reconstruction methods that may increase MRI energy usage while reducing the need for a scarce resource like gadolinium [31].

These complex scenarios require comprehensive analysis of the circular economy of imaging equipment and pharmaceuticals, which includes their production, energy use and disposal. Specific issues that need to be addressed in addition to fossil fuel emissions are anthropogenic gadolinium contamination in ground and surface waters, mineral extraction and critical mineral availability [32].

## 5 Education and Climate literacy

A well‐informed and engaged radiology workforce is essential to steward a transition to environmentally sustainable medical imaging. Leadership in this area begins with understanding the health harms of the climate crisis and the co‐benefits of reducing air pollution, biodiversity loss and climate change. This is largely understood by healthcare professionals; however, a variety of barriers preclude engagement [33].

Integration of planetary health education into Diagnostic Radiology curricula including continuing medical education will ensure that radiologists acquire the necessary knowledge and skills to engage in mitigation and adaptation of medical imaging to the climate crisis, and a commitment to lifelong learning will allow rapid response and leadership as it evolves. Radiologists should be versed in the changing patterns of vector borne disease, cardiopulmonary and renal disease, cancer incidence and survivorship following extreme weather events, including increasing respiratory illnesses, heat related health emergencies and increased risk of injury and prevalence of mental health crises [5, 6]. Cross‐disciplinary collaboration with all our partners will be essential.

There should be a focus on collaboration and avoidance of duplication. Radiology conferences and journals, augmented by social media and open networks such as Radiologists for a Sustainable Future (@Rads4SF) allow for rapid distribution of successful strategies and crowdsourcing of efforts expanding micro (local) efforts to the macro (national and global) level. Cross‐national and multi‐society sharing of educational materials, sustainability quality improvement projects, research and planetary health resources will also encourage curricular innovation and research. Radiology societies need to promote highly visible educational sessions on climate change and environmental sustainability at imaging conferences and through on‐line platforms.

Tracking progress on reducing emissions and waste and sharing successful initiatives will increase confidence in organisational commitment and highlight what is possible with many contributing to the effort.

The healthcare community as trusted voices on health have tremendous potential to influence social and public policy in support of decarbonization in our workplaces and communities, and in shaping global climate policy [34].

## 6 Operational sustainability and appropriate use: toolkit

Management of resources, including energy, materials, equipment and workforce, is essential for a sustainable imaging practice. While decisions on sources of electricity and energy or waste management at an organisational level may be out of the direct control of radiology departments, radiology leaders can advocate for sustainable practices. Working in coordination with operations and quality and safety departments, radiologists should consider sustainability and the circularity of the supply chain when purchasing equipment and eliminating waste [35, 36]. The use of smart monitoring tools can help to mitigate energy consumption related to heating, ventilation and air‐conditioning (HVAC), lighting and machine‐on time [35].

Reducing unnecessary, low‐value imaging is paramount and requires broad engagement including radiologists in collaboration with referring clinicians and patient partners. New digital innovations hold the promise of AI‐assisted decision‐support tools to help guide appropriate use of imaging and adherence to guidelines for screening and follow up. Opportunistic data capture provides an opportunity to capture relevant incidental screening information and data [37]. The development of integrated and interoperable electronic medical records and advances in image and data sharing can eliminate redundancies. Guidelines for appropriate use and resource allocation must prioritise value‐based care and be mindful of the various resource constraints, including underuse of resources, in countries and communities with limited access. Decision support tools and appropriate use criteria must be easy to access and apply and should be integrated into ordering protocols.

## 7 Preparedness: Adapting to climate change and preparing for climate emergencies

In parallel to mitigation strategies, adaptation strategies are needed to build resiliency to current and future impacts of the climate crisis [35, 36]. Environmental exposures and adverse weather can lead to higher demand for healthcare, including unpredictable swings in the demand for imaging [5, 25]. Such events also pose a risk to infrastructure and to the health, safety and availability of the workforce which in turn can impact capacity and productivity.

### 7.1 Recognising operational vulnerability and preparing for crisis

**FIGURE 2:**
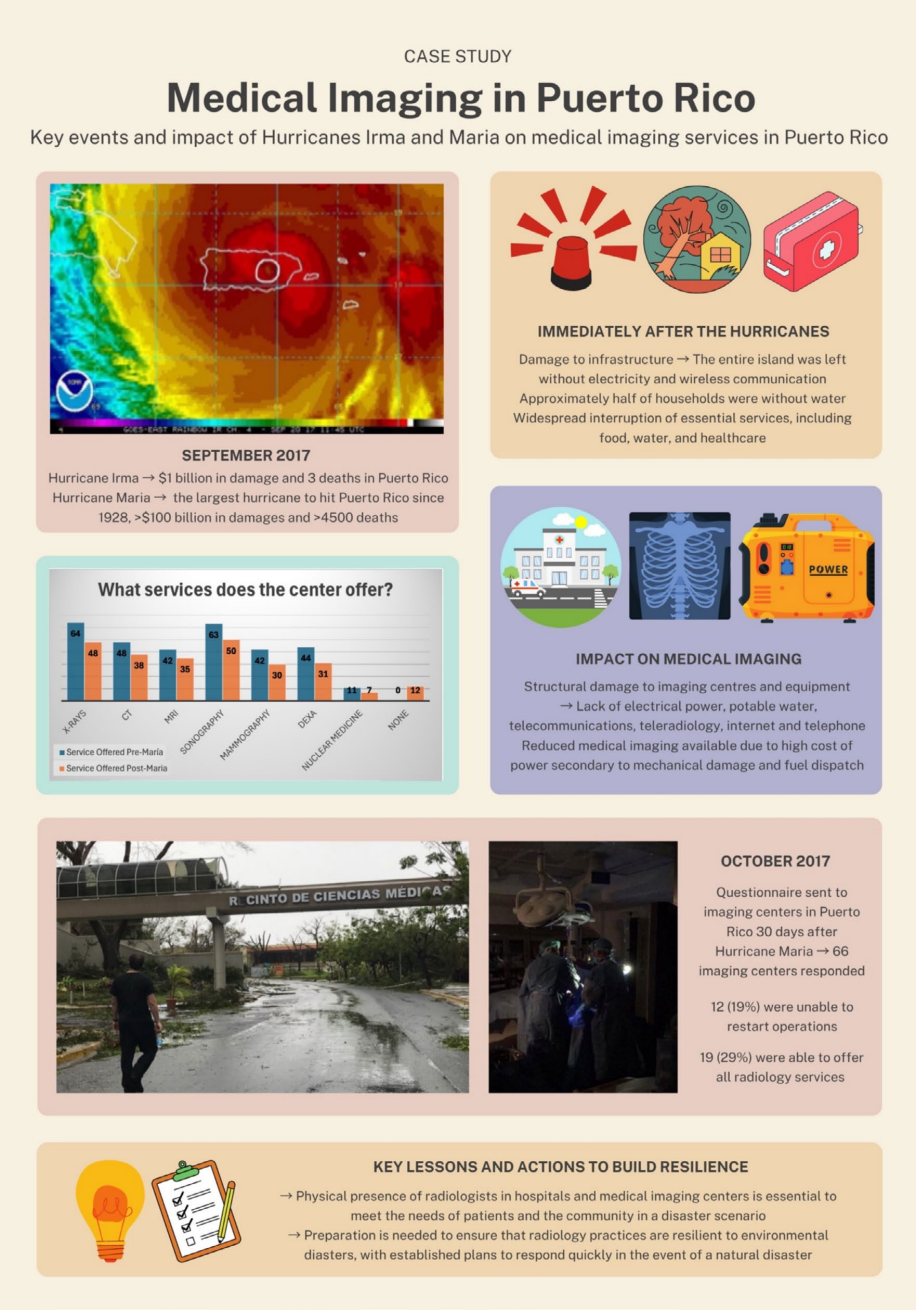
Case study medical imaging in Puerto Rico: key events and impact of hurricanes irma and maria on medical imaging services in Puerto Rico.

Climate emergencies can disrupt imaging operations due to failed equipment, infrastructure damage, strains on the energy grid, or inadequate staffing. Radiology departments are susceptible to flooding, extreme temperatures and power failures [3]. These vulnerabilities can lead to delayed care as well as financial strain. Development of disaster management protocols to prepare for extreme weather events, including potential work‐force shortages and surges in imaging volumes is essential (Table 2) [4]. A case study describes the impact of climate change in radiology in Puerto Rico (Figure 2 and Table 3).

**TABLE 2:** Preparing radiology departments for climate resilience.

Goal	Action
Good teamwork	IT and Operations teams must be coordinated and work together [35]
Infrastructure upgrade	Infrastructure must be upgraded to ensure minimal damage to imaging equipment in the event of flooding, storms, extreme temperature and power outages [9, 38]
Technology system redundancy	Minimise data loss through building redundancy in technological systems and data storage with back‐up power sources [9, 39] Back up communications systems
Human resource resilience	Identify resilience for imaging staffing, service coverage, reporting, information systems team
Operational System redundancy	Identify alternate energy sources, supply chains and infrastructure when disrupted or damaged [8]
Clinical emergency planning	Identify alternate patient access routes, staff access and outline triage plans

**TABLE 3:** Findings of a questionnaire sent to 66 imaging centres in Puerto Rico by Sociedad Radiológica de Puerto Rico (SOCRAD) 30 days after Hurricane Maria in 2018. Imaging Centres reported a decrease in the number of modalities offered after Hurricane Maria and 12 centers were not able to offer any imaging services.

Imaging modality offered	Service offered per‐Maria	Service offered post‐Maria	Difference
Radiographs	64	48	25%
CT	48	38	21%
MRI	42	35	17%
Sonography	63	50	21%
Mammography	42	30	29%
DEXA	44	31	30%
Nuclear medicine	11	7	36%
No imaging services available	0	12	18%

The key focus of disaster planning and resiliency measures is to ensure continuity of care during crises. Investment in climate resilient facilities through infrastructure upgrades is needed [36]. Resiliency requires flexibility and coordination, supply chain and workforce planning, as well as operational and data system redundancies.

### 7.2 At the time of crisis: workforce and patient care

Climate related environmental exposures, including air pollution, wildfire smoke and extreme heat, are associated with increased health system utilisation [8]. Workers too are vulnerable to climate impacts and may themselves become patients. A climate emergency may impact patients' ability to access imaging sites and services or workers' ability to provide those services.

Departments should take steps to reduce both patients' and workers' vulnerability to climate impacts. Healthcare institutions must take the lead on climate solutions and identify and collaborate with communities that are disproportionately at risk from climate‐related harm. Preventive measures and investments that aim to improve the co‐ordination of community resilience and preparedness, health, well‐being and equity will be necessary to create truly resilient healthcare systems.

## 8 Patient‐centred perspective and collaboration

Connecting the concept of environmental sustainability with improved patient outcomes may help to frame the issue in terms of patient‐centred care. For example, reducing the environmental impact of healthcare will contribute to improved long‐term public health outcomes. Efforts to identify disease at early stages, avoidance of low‐value investigations, the development of community based diagnostic hubs and diagnostic outreach for remote communities can increase access, improve patient pathways, reduce emissions from patient travel by co‐ordinating appointments as well as providing more accessible, community‐based imaging services and reduce the environmental impact of services.

Listening to the patient's voice and retaining patient trust is critically important if changes to a care pathway are being made to avoid any perception that we might be ‘short‐changing’ patient care when we are ensuring guideline‐compliant care that is most appropriate for their medical condition. Indeed, identifying strong patient advocates for environmental sustainability in healthcare is an important resource at this time of transition.

## 9 Conclusion

Transformation of radiology to more environmentally sustainable practices is urgent. We need research to better understand the current state of sustainability in the practice and to help prioritise mitigation actions with the highest impact. At the same time regional and global disparities in access to care, which stand to be exacerbated by climate change, must be addressed. Despite current knowledge gaps, we are ready to lead the change and transform to sustainable radiology, working closely with industry and patients (consumers) and preparing for potential climate crises. As healthcare professionals and radiology societies, working together as a global team, we must make our trusted voices heard by policy makers to influence a revolution in the way we work.

## Ethics Statement

Institutional Review Board approval was not required.

## Consent

Written informed consent was not required.

## Conflicts of Interest

Andrea G. Rockall: none in relation to this article. Bibb Allen: none in relation to this article. Maura J. Brown: none in relation to this article. Tarek El‐Diasty: none in relation to this article. Jan Fletcher: none in relation to this article. Rachel F. Gerson: none in relation to this article. Stacey Goergen: none in relation to this article. Amanda P. Marrero González: none in relation to this article. Thomas M. Grist: none in relation to this article. Kate Hanneman: Associate Editor Canadian Association of Radiologists Journal, Radiology, Radiology: Cardiothoracic Imaging, and Journal of Cardiovascular Magnetic Resonance. Christopher P. Hess: none in relation to this article. Evelyn L.M. Ho: none in relation to this article. Dina H. Salama: none in relation to this article. Julia Schoen: none in relation to this article. Sarah Sheard: none in relation to this article. The authors of this manuscript declare no relationships with any companies, whose products or services may be related to the subject matter of the article.

## Statistics and Biometry

No complex statistical methods were necessary for this paper.

## Methodology

Review of literature and expert opinion gathering for society position paper.

## Guarantor

The scientific guarantor of this publication is Andrea G. Rockall.

## Data Availability

The data that support the findings of this study are available from the corresponding author upon reasonable request.
